# New insights in the Fontan circulation: 4-dimensional respiratory- and ECG-triggered phase contrast magnetic resonance imaging

**DOI:** 10.1186/1532-429X-15-S1-O38

**Published:** 2013-01-30

**Authors:** Christopher Hart, Dominik Daniel Gabbert, Inga Voges, Michael Jerosch-Herold, Ana Andrade, Minh Pham, Traudel Hansen, Hans-Heiner Kramer, Carsten Rickers

**Affiliations:** 1 Pediatric Cardiology, Universitaetsklinik Kiel, Kiel, Germany; 2 Radiology, Brigham and Women's Hospital, Boston, MA, USA

## Background

Evaluation of blood flow characteristics in total cavo-pulmonary connection (TCPC) with CMR remains difficult due to its strong modulation by respiration, and is not yet entirely understood. New approaches using 4D phase contrast magnetic resonance imaging (4D PC MRI) are promising and can contribute to the understanding of hemodynamics in the Fontan Circulation. Our objective was to compare flow, velocities, wall shear stress (WSS) and circulation in the TCPC using respiratory- and ECG triggered 4D PC MRI.

## Methods

10 children with hypoplastic left heart syndrome were evaluated after surgical completion of the Fontan circulation (TCPC with lateral intra-atrial tunnel) in a single center. In all patients one respiratory- (80 -100 phases) and one ECG-triggered (30 phases) 4D PC MRI covering the whole thorax, voxel size ranging from isotropic 1.5 to 2.0 mm were acquired during a single CMR examination with a custom-made electronic respiratory triggering compatible with our scanner. Dedicated commercial and custom software was used for further analysis of flow, velocity, WSS and circulation.

## Results

Respiratory-triggered acquisitions revealed significantly higher maximum and lower minimum flow, maximum and minimum velocity, maximum WSS, and maximum circulation in the inferior vena cava and tunnel, compared to ECG-triggered 4D PC MRI. Flow, velocity, WSS and circulation in the superior vena cava, and also the flow distribution to the left and right pulmonary arteries showed no differences between the two acquisition modes.

## Conclusions

Respiration-triggered 4D PC MRI of the TCPC avoids averaging of flow, velocity, and WSS over the respiratory cycle, resulting in significant differences to solely ECG-triggered acquisitions. This study suggests that hemodynamics in the TCPC are mainly dependant on respiration, while ventricular function causes only minor modulation of flows in the TCPC connection. This may have potential clinical implications for judging the quality of a TCPC, for example in the failing Fontan, by 4D PC MRI. The data from the respiratory triggered sequences can further add to the understanding of hemodynamics and fluid mechanics in the Fontan circulation.

## Funding

None.

